# Effects of hydrogen-rich water on blood uric acid in patients with hyperuricemia: A randomized placebo-controlled trial

**DOI:** 10.1016/j.heliyon.2024.e36401

**Published:** 2024-08-15

**Authors:** Fenglin Wu, Jun Ma, Junli Xue, Xue Jiang, Jinyu Liu, Jiashuo Zhang, Yazhuo Xue, Boyan Liu, Shucun Qin

**Affiliations:** a Shandong Provincial Key Medical and Health Laboratory of Hydrogen Biomedical Research & Key Laboratory of Major Diseases and Hydrogen Medical Translational Applications in Universities of Shandong Province, Taishan Institute for Hydrogen Biomedical Research, The Second Affiliated Hospital of Shandong First Medical University, Tai'an, China; b College of Nursing, Shandong First Medical University and Shandong Academy of Medical Sciences, Tai'an, China; c Shandong Cancer Hospital and Institute, Shandong First Medical University and Shandong Academy of Medical Sciences, Jinan, China; d Taishan Vocational College of Nursing, Tai'an, China

**Keywords:** Blood uric acid, Hyperuricemia, Hydrogen-rich water, Dose-effect, Time-effect

## Abstract

**Background:**

Consumption of hydrogen-rich water (HRW) has been shown to have anti-inflammatory and metabolic-modulatory benefits.

**Objective:**

A randomized, placebo-controlled trial was conducted to assess the potential blood uric acid-lowering effects of HRW consumption with different doses (low and high doses) and duration (4 and 8 weeks) in patients with hyperuricemia.

**Methods:**

The Placebo group consumed three bottles of ordinary drinking water (330 mL per bottle), the Low-HRW group consumed two bottles of HRW (330 mL per bottle, H_2_ ≥ 4.66 mg/L) and a bottle of ordinary water, and the High-HRW group consumed three bottles of HRW daily for 8 weeks. The primary outcome was the blood uric acid levels following different time points (4 and 8 weeks) compared to baseline.

**Results:**

A total of 100 participants completed the entire trial (32 in Placebo, 35 in Low-HRW, and 33 in High-HRW groups). The high-dose of HRW was more effective than low-dose HRW in controlling blood uric acid. Following an 8-week period, the High-HRW group exhibited a significant reduction in blood uric acid levels compared to the baseline (488.2 ± 54.1 μmol/L to 446.8 ± 57.1 μmol/L, *P* < 0.05).

**Conclusion:**

As a rather safe agent, the prolonged consumption of HRW may be feasible in the management of hyperuricemia.

**Clinical trial registration:**

chictr.org.cn, identifier ChiCTR2200066369.

## 1 Introduction

Hyperuricemia, a metabolic disorder resulting from impaired purine metabolism, exhibits a higher incidence among middle-aged and elderly males, as well as postmenopausal females. In China, the prevalence of hyperuricemia among adults is estimated to be 14.0 %, including 24.4 % in males and 3.6 % in females, and it continues to increase annually [1,2].

Lifestyle modifications, such as adopting a healthy diet, limiting alcohol consumption, and engaging in regular exercise, have been empirically demonstrated to be efficacious in the prevention and management of hyperuricemia [3,4]. However, when lifestyle modifications cannot achieve the expected effect, specific medications become necessary.

The existing drugs for the treatment of hyperuricemia include uricostatic agents (allopurinol and febuxostat), uricosuric agents (probenecid, benzbromarone, and losartan), and uricolytic agents (plegoticase and rasburicase) [5]. Nevertheless, the majority of these drugs intended to reduce uric acid level are associated with adverse effects, including increased risks of gastrointestinal disorders, cardiovascular diseases, hepatic impairment, and renal dysfunction [5,6]. For instance, allopurinol is a widely used drug of reducing uric acid level, but the occurrence of allopurinol-induced severe cutaneous adverse drug reactions has been reported to range from 3 to 4.68 cases per 1000 new users [6,7]. Consequently, it is imperative to explore a safe and effective supplementary treatment to assist patients in reducing uric acid level while mitigating adverse effects.

Multiple studies have demonstrated a close association between hyperuricemia and oxidative damage caused by reactive oxygen species, and there was a strong correlation between inflammation and uric acid levels across the entire spectrum from hyperuricemia to gout [8,9]. Meanwhile, hyperuricemia has been associated with various diseases including acute and chronic nephropathy, obesity, metabolic syndrome, diabetes, cardiovascular diseases, and hypertension [[10], [11], [12]].

Molecular hydrogen (H_2_) has been proven to be an anti-oxidative and anti-inflammatory agent [[13], [14], [15]]. Clinical studies have demonstrated that H_2_ could play a role in the management of obesity, diabetes, cardiovascular diseases, *et*c [[16], [17], [18], [19], [20]]. These findings imply the potential utility of H_2_ in the treatment of hyperuricemia.

There are various routes for H_2_ administration such as H_2_-rich water (HRW) drinking, H_2_ gas inhalation, and H_2_ water bath [21,22]. HRW refers to regular water that contains dissolved H_2_, and HRW drinking is convenient and extremely bio-safe. A previous randomized placebo-controlled clinical study revealed that a 3-month HRW drinking could exhibit therapeutic benefits in male patients with hyperuricemia [23]. However, the time-effect and dose-effect of HRW are still required for enhanced clinical efficacy.

In this study, we conducted a randomized, placebo-controlled trial to assess the effectiveness of various doses and durations of HRW consumption on patients with hyperuricemia.

## 2 Materials and methods

### 2.1 Study design

This study consisted of an 8-week randomized, placebo-controlled, parallel-design trial conducted from May to July 2023. After obtaining informed consent, all participants were randomly allocated to the Placebo group, the Low-HRW group, or the High-HRW group. The intervention was single-blind. The study followed the recommendations of the Consolidated Standards of Reporting Trials (CONSORT) statement for reporting randomized controlled trials. Written informed consent was obtained from all participants. The trial was approved by the Ethical Committee of the Second Affiliated Hospital of Shandong First Medical University (No.2022-122) and was registered at the Chinese Clinical Trial Registry (chictr.org.cn) with the registration number ChiCTR2200066369 (registered on: December 2, 2022). All procedures were carried out in accordance with the Helsinki Declaration.

### 2.2 Participants

Participants were recruited from Longkou City, Shandong, China. Adult participants with baseline blood uric acid levels >420 μmol/L for males and >360 μmol/L for females were enrolled [24,25]. Exclusion criteria included individuals taking medications that could potentially decrease uric acid levels, those with gout arthritis, with a medical history of severe conditions such as heart failure, malignant tumors, or organ transplant, as well as women during pregnancy or lactating. The criteria for discontinuation encompassed withdrawal of consent, non-compliance, or other medical factors that required the termination of the intervention. Randomized control assignment was performed by an investigator utilizing the R program (1: 1: 1). Each number was placed in a separate, opaque envelope kept by the investigator; each envelope contained the treatment allocation card (experimental group or placebo group). All the investigators knew the assignment and the intervention plan, but the participants did not.

### 2.3 Intervention

Participants of the Placebo group were instructed to consume three bottles of placebo water (ordinary drinking water with the same appearance as HRW, 330 mL per bottle) daily. The Low-HRW group consumed two bottles of HRW (330 mL per bottle) and one bottle of placebo water daily. The High-HRW group consumed three bottles of HRW daily. The HRW and placebo water are packaged in aluminum cans and supplied by the Beijing Huoliqingyuan Co., Ltd. (Beijing, China). Participants were instructed to consume one bottle of water within 10 min in the morning, noon, and evening, respectively. All participants were instructed to adhere to their regular dietary, pharmaceutical, and lifestyle practices. The H_2_ concentration of HRW was determined by a Clark-type H_2_ microsensor (Unisense, Aarhus N, Denmark) in our laboratory by the method previously reported [22], and the H_2_ concentration was ≥4.66 mg/L. By calculation, the daily H_2_ intake doses were about 3.08 mg and 4.61 mg in the Low-HRW group and High-HRW group, respectively.

### 2.4 Outcomes

The primary outcome of this study was the blood uric acid level, and the secondary outcomes included the proportion of participants who achieved a reduction of at least 10 % in blood uric acid after 4 and 8 weeks, as well as the plasma biochemical parameters. The blood uric acid was detected after overnight fasting in participants' fourth finger using a portable uric acid detector (Sannuo Biosensing Co., Ltd., Hunan, China) by a trained technician, and the average of two consecutive measurements was taken. Fasting venous blood samples from median cubital vein were collected into EDTA tubes and subsequently centrifuged (15 min at 1300 g) to obtain plasma. Alkaline phosphatase (ALP), aspartic aminotransferase (AST), alanine aminotransferase (ALT), β2-microglobulin (β2-MG), creatinine (Cr), blood urea nitrogen (BUN), total cholesterol (TC), HDL-cholesterol (HDL-C), LDL-cholesterol (LDL-C), and triglyceride (TG) and glucose (GLU) were analyzed by an automatic biochemical analyzer (HITACHI 7080).

### 2.5 Sample size estimation

The sample size was estimated using the G-Power software, with a 95 % confidence interval, a test power of 85 %, and a 15 % probable drop, and the sample size was calculated to be 114 for three groups.

### 2.6 Statistical analysis

The data were presented as mean and standard deviation (mean ± SD), median (Quantile 1 and Quantile 3), or percentage. The presence of skewness, outliers, and systematic missing data was evaluated. The normal distribution of the data was assessed using the Shapiro–Wilk test. The blood uric acid levels were analyzed using repeated-measures analysis of variance (ANOVA) followed by Bonferroni's test. The ANOVA, unpaired *t*-test, and paired t-tests were used to detect inter and intra-group differences in data that adhered to a normal distribution. For data that did not conform to a normal distribution, the Mann-Whitney *U* test (unpaired) and Wilcoxon signed rank test (paired) were utilized to compare differences. To determine differences between groups of categorical data (eg, proportion with underlying diseases or proportion with ≥10 % decline in blood uric acid), Pearson χ^2^ test or Fisher exact test were used as applicable. Statistical analyzes were performed by SPSS 26.0 (SPSS Inc., Chicago, IL, USA). *P* < 0.05 was considered as significantly different in statistics.

## 3 Results

### 3.1 Participants

**Fig. 1:**
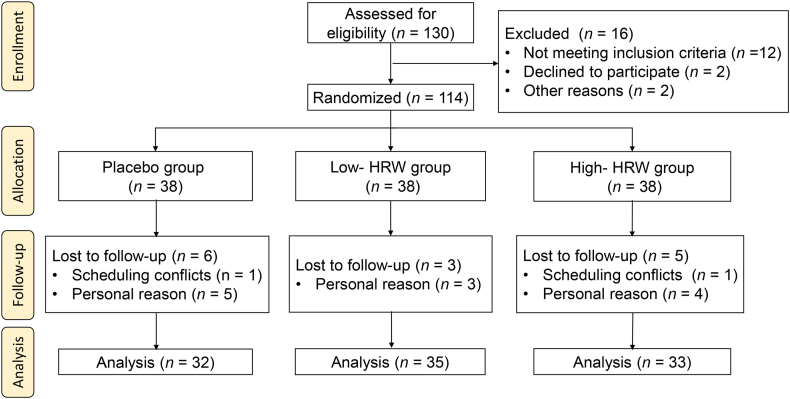
Trial flowchart.

A total of 130 participants agreed to participate in this study, and 114 participants were recruited according to the inclusion and exclusion criteria. These participants were then randomly assigned to the Placebo group (*n* = 38), the Low-HRW group (*n* = 38), or the High-HRW group (*n* = 38). During the follow-up, 6 participants in the Placebo group (One participant was dropped out due to scheduling conflicts and five participants concerned about personal reasons), 3 participants in the Low-HRW group (Three participants were dropped out due to personal reasons), and 5 participants in the High-HRW group (One participant was dropped out due to scheduling conflicts and four participants concerned about personal reasons) withdrew their participation. Finally, a total of 32 participants from the Placebo group, 35 participants from the Low-HRW group, and 33 participants from the High-HRW group successfully completed the 8-week follow-up study (Fig. 1). Upon analysis, it was determined that there were no significant differences in baseline characteristics among the three groups for participants who completed the trial (*P* > 0.05, Table 1). Furthermore, no adverse effects were reported throughout the entire duration of the study.

**Table 1:** Baseline characteristics of participants.

Characteristics	Placebo group (*n* = 32)	Low-HRW group (*n* = 35)	High-HRW group (*n* = 33)	*P*
Male, No. (%)	26 (21.25)	30 (85.71)	27 (81.82)	0.867
Female, No. (%)	6 (18.75)	5 (14.29)	6 (18.18)	0.867
Age, years	35.25 ± 8.08	34.23 ± 8.57	35.36 ± 8.75	0.831
Weight, kg	79.91 ± 16.50	85.23 ± 15.21	83.94 ± 19.78	0.428
Body mass index	26.72 ± 4.70	28.16 ± 4.57	27.44 ± 4.94	0.466
SBP, mmHg	125.78 ± 13.16	126.60 ± 21.89	128.63 ± 11.12	0.766
DBP, mmHg	79.98 ± 9.56	81.26 ± 8.88	79.82 ± 8.76	0.768
Smoking, *n* (%)	11 (34.38)	11 (31.43)	10 (30.30)	0.955
Alcohol drinking, *n* (%)	8 (25.00)	7 (20.00)	7 (21.21)	0.878
Uric acid, μmol/L	486.4 ± 71.1	484.8 ± 64.6	488.2 ± 54.1	0.975
Underlying diseases				
Fatty liver, *n* (%)	7 (21.88)	5 (14.29)	4 (12.12)	0.531
Hypertension, *n* (%)	2 (6.25)	1 (2.86)	2 (6.06)	0.77
gout, *n* (%)	0 (0)	1 (2.86)	1 (3.03)	0.618
Diabetes, *n* (%)	0 (0)	1 (2.86)	1 (3.03)	0.618

Age, weight, body mass index, SBP, DBP, and uric acid are presented as the mean ± SD. Intergroup differences were tested by Analysis of Variance (age, weight, body mass index, SBP, DBP, uric acid) or the Kruskall-Wallis test (sex, smoking, alcohol drinking, underlying diseases).

### 3.2 Blood uric acid

**Fig. 2:**
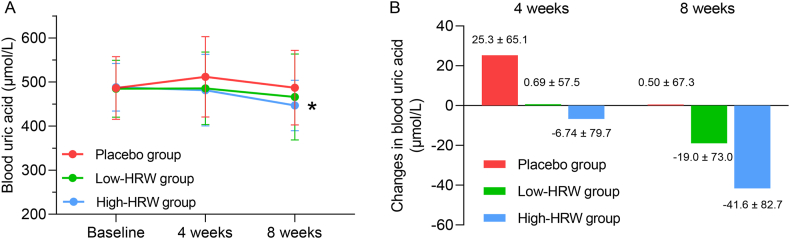
A: Blood uric acid at baseline and after 4 weeks and 8 weeks of intervention; B: Changes in blood uric acid compared to the baseline. Data are represented as the mean ± SD; *P < 0.05 compared to the Baseline.

The blood uric acid levels were assessed prior to the intervention, as well as at 4 weeks and 8 weeks following the intervention. As shown in Fig. 2A and Table S1, there were no statistically significant differences in blood uric acid levels between the Placebo and Low-HRW groups at various time points, although a slight but not significant decrease was observed at 8 weeks in the Low-HRW group compared to baseline (P > 0.05). In contrast, the High-HRW group exhibited a tendency towards decreased blood uric acid levels at 4 weeks (P > 0.05), with a significant decrease observed at 8 weeks compared to baseline (488.2 ± 54.1 μmol/L to 446.8 ± 57.1 μmol/L, P < 0.05).

The changes in blood uric acid levels are depicted in Fig. 2B and Table S1. After 4 weeks, the average uric acid level in the Low-HRW group exhibited minimal variation compared to the baseline, and it experienced a reduction of 6.74 μmol/L in the High-HRW group. After a period of 8 weeks, the average uric acid level decreased by 19.0 μmol/L in the Low-HRW group and 41.6 μmol/L in the High-HRW group.

We further analyzed the number and percentage of participants with over 10 % decline in blood uric acid level (Table 2). In the Low-HRW group, 7 out of 35 participants (39.4 %) and 13 out of 35 participants (37.1 %) demonstrated a ≥10 % decrease in blood uric acid levels after 4 and 8 weeks, respectively. Meanwhile, in the High-HRW group, 8 out of 33 participants (24.2 %) and 13 out of 33 participants (39.4 %) exhibited a ≥10 % decrease in blood uric acid after 4 and 8 weeks, respectively. There were no significant differences in either the Low-HRW group and the High-HRW group compared to the Placebo group or the Low-HRW group compared to the High-HRW group (*P* > 0.05).

**Table 2:** Participants exhibiting a reduction of more than 10 % in blood uric acid levels.

	Placebo group (*n* = 32)	Low-HRW group (*n* = 35)	High-HRW group (*n* = 33)	*P*^a^	*P*^b^	*P*^*c*^
4 weeks	3 (9.38 %)	7 (20 %)	8 (24.2 %)	0.38	0.19	0.77
8 weeks	7 (21.9 %)	13 (37.1 %)	13 (39.4 %)	0.19	0.18	1.00

Data are presented as numbers (percentages). ^a^Low-HRW group vs. Placebo group. ^b^High-HRW group vs. Placebo group. ^c^Low-HRW group vs. High-HRW group.

### 3.3 Blood assays

Plasma biochemical indicators of all participants were examined before and after the trial. As shown in Table 3, for the liver function indicators, the ALP levels were significantly lower in the Low-HRW group and High-HRW group compared to the baseline after an 8-week intervention, while it displayed a significant increase in the Placebo group. However, no statistically significant differences were observed in the AST and ALT levels among the three groups (P > 0.05).

**Table 3:** Plasma biochemical parameters at baseline and after the 8-week intervention.

	Placebo group (*n* = 32)		Low-HRW group (*n* = 35)		High-HRW group (*n* = 33)	
	Baseline	8 weeks	Baseline	8 weeks	Baseline	8 weeks
ALP, U/L	78.9 ± 16.2	79.2 ± 17.3*	74.8 ± 20.4	71.9 ± 16.0*	76.4 ± 16.1	75.7 ± 18.7*
AST, U/L	19.0 (15, 31.3)	21.0 (16.3, 28.8)	19.0 (16.0, 24.0)	21.0 (17.0, 25.5)	21.0 (15.0, 30.5)	22.0 (18.5, 31.0)
ALT, U/L	29.5 (20.5, 49.3)	29.5 (17.3, 44.8)	30.0 (21.0, 36.0)	29.0 (20.0, 38.0)	26.0 (18.5, 50.0)	27.0 (21.0, 44.0)
β2-MG, mg/L	1.70 (1.58, 2.00)	1.81 (1.63, 2.09)	1.55 (1.33, 1.94)	1.66 (1.36, 1.84)	1.70 (1.44, 1.87)	1.66 (1.40, 1.85)
BUN, mmol/L	5.07 ± 0.91	5.29 ± 1.09*	4.91 ± 0.95	5.19 ± 1.16*	4.80 ± 0.76	4.90 ± 1.12*
Cr, μmol/L	74.5 (66.3, 88.0)	71.6 (63.8, 84.3)	75.0 (67.0, 82.0)	77.2 (72.0, 81.4)	74.0 (65.6, 83.0)	75.1 (66.3, 85.6)
TC, mmol/L	4.37 (3.83, 5.26)	4.44 (3.98, 5.31)	4.56 (3.91, 5.19)	4.50 (4.01, 4.91)	4.55 (4.09, 5.41)	4.65 (4.34, 5.39)
LDL-C, mmol/L	2.71 (2.28, 3.34)	2.75 (2.11, 3.15)	2.95 (2.41, 3.30)	2.85 (2.21, 3.11)	2.77 (2.34, 3.31)	2.78 (2.36, 3.18)
HDL-C, mmol/L	1.09 (0.98, 1.24)	1.02 (0.95, 1.15)*	1.11 (0.96, 1.35)	1.05 (0.94, 1.27)*	1.17 (1.07, 1.37)	1.15 (1.00, 1.30)*
TG, mmol/L	1.81 (1.18, 2.81)	1.98 (1.04, 2.43)	1.47 (0.99, 2.26)	1.73 (1.08, 2.64)	1.61 (1.05, 2.19)	1.47 (1.10, 2.43)
GLU, mmol/L	5.09 (4.60, 5.45)	5.33 (5.05, 5.60)*	5.10 (4.79, 5.32)	5.29 (4.99, 5.67)*	5.06 (4.80, 5.29)	5.24 (5.00, 5.47)*

Data are median (Quantile 1, Quantile 3) or mean ± SD. *P < 0.05 vs. baseline in the same group by paired *t*-test or Wilcoxon signed rank test.

For the renal function indices, the alterations in β2-MG and Cr did not exhibit statistical significance (*P* > 0.05) in both the Low-HRW group and High-HRW group. Nevertheless, the levels of BUN demonstrated a significant increase in all three groups after 8 weeks compared to the baseline. The lipid-related indices, including TC, LDL-C, and TG, did not display any statistically significant differences after the 8-week intervention. However, the levels of HDL-C noticeably decreased in all three groups following the intervention. Additionally, the GLU levels exhibited a marked increase in all three groups after the intervention.

## 4 Discussion

In the current randomized placebo-controlled study, it was observed that an 8 weeks High-HRW intervention resulted in a significant reduction in uric acid levels compared to baseline.

H_2_ is acknowledged as a relatively safe agent. In the food industry, H_2_ has obtained official approval as a food additive due to its non-toxic properties [16,26]. A clinical trial has demonstrated that the healthy adults experienced no adverse effects when exposed to 2.4 % H_2_ gas through inhalation for a duration of up to 72 h [27]. Thus, H_2_ is well suited for use in adjuvant therapy.

HRW consumption is feasible for daily use. Compared with other H_2_-supply routes such as inhaling H_2_ gas, taking a H_2_-water bath, or stimulating intestinal microbiomes to produce H_2_, the consumption of bottled HRW ensures an accurate H_2_-dose. Previous clinical studies have demonstrated the potential benefits of HRW consumption in patients with type 2 diabetes [28], metabolic syndrome [29], unstable angina [17], etc. A six-month study found that the consuming of HRW reduced the level of uric acid in the plasma of healthy rats [30]. Meanwhile, a previous clinical trial assessed that the consumption of HRW could decrease the uric acid levels among individuals with hyperuricemia [23]. However, few studies have been conducted to investigate the time effect and dose effect of H_2_ intake. In the current study, low and high doses of HRW were employed, and the effects at different times of 4 weeks and 8 weeks were investigated.

The solubility of H_2_ in water is approximately 1.6 mg/L under standard conditions (20 °C, 101.325 kPa). The utilization of micro and nanobubbles technologies has been shown to enhance the solubility of H_2_ in water. In the previous clinical studies, the H_2_ concentrations in HRW varied from 0.4 to 1.6 mg/L [29,31], and it resulting in significant differences in the daily intake of H_2_. In our current study, the concentration of H_2_ in HRW was measured as 4.66 mg/L.

The optimal duration for the HRW intervention remains unexplored. For the intervention of metabolic syndrome, the duration of intervention ranging from 8 to 24 weeks [29,32,33], and HRW consuming effectively regulated plasma lipid indices. For patients with non-alcoholic fatty liver disease, HRW intervention durations ranged from 4 to 8 weeks [19,34,35]. In order to assess the intervention effect over time, the current study examined blood uric acid levels after 4 and 8 weeks of HRW consumption. The results of our trial indicate a significant reduction in uric acid levels following an 8-week high-dose HRW intervention. In contrast to pharmacological interventions., the consumption of HRW appears to require a long-term administration for the attainment of clinical efficacy. Considering its remarkable safety profile, HRW is suited as an adjuvant therapy for metabolic disorders.

Hyperuricemia, a metabolic disorder caused by elevated levels of uric acid in the bloodstream due to disrupted purine metabolism, has been found to be associated with inflammatory disorders in previous studies [8,36]. There have studies indicated that the levels of inflammatory markers, including interleukin-6 (IL-6), tumour necrosis factor-α (TNF-α) and transforming growth factor-β1 (TGF-β1), are markedly elevated in individuals with hyperuricaemia in comparison to those in a healthy state [37]. The levels of IL-6, TNF-α, and TGF-β1 may exacerbate renal tubular and interstitial damage, which may in turn lead to impaired uric acid excretion [37]. Oxidative stress plays a pivotal role in the pathogenesis, progression and regression of hyperuricaemia. Xanthine oxidoreductases (XORs) are pivotal enzymes in uric acid production [38,39]. It has been demonstrated that in humans, XORs may be implicated in the aetiology of metabolic disorders such as hyperuricaemia and metabolic syndrome through oxidative stress, XORs-derived reactive oxygen species, and uric acid-induced inflammatory responses [40,41]. Extensive basic and clinical studies have demonstrated that H_2_ possesses the capacity to inhibit inflammatory responses, suggesting its potential benefits in the management of hyperuricemia. The potential mechanisms involved in the antioxidant and anti-inflammatory effects of H_2_ have not yet been completely elucidated. Ohsawa et al. found that H_2_ selectively reduced hydroxyl radical in cultured cells [13]. Jin et al. proposed that ferroporphyrin may be a biological target of H_2_, through which hydroxyl radical is reduced into H_2_O [42]. The mechanisms of HRW consumption on regulating oxidative stress and inflammatory responses, and thereby reducing blood uric acid levels require further inquiry.

Studies have showed a clear relationship of increased uric acid levels with various pathological conditions such as metabolic syndrome, obesity, hypertension, and cardiovascular events [[10], [11], [12]]. Previous clinical studies indicated that consumption of HRW might exert beneficial effect on multiple metabolic diseases. A placebo-controlled trial demonstrated that supplementation with a high-concentration of HRW produced via H_2_-producing tablets favorably modulated fatty acid and glucose metabolism, and improved inflammation and redox homeostasis in subjects with metabolic syndrome [33]. In patients with type 2 diabetes mellitus, it is noted that supplementation with HRW decreased the levels of oxidized LDL, free fatty acids, and urinary 8-isoprostanes, and may have a beneficial role in the insulin resistance [28]. Another placebo-controlled clinical study suggested that H_2_ may have potentially beneficial impact on glucose metabolism by modifying the gut microbiota of individuals with impaired fasting glucose [20]. For the regulation of lipid metabolism, it is suggested that HRW have beneficial lipid-lowering effects and can improve high-density lipoprotein function in patients with hypercholesterolemia. Meanwhile, several studies have shown that the consumption of HRW could have advantageous effects on diverse biomarkers for liver function and regulate lipid levels in patients with non-alcoholic fatty liver disease [19,34,43]. The ameliorative effect of HRW on these diseases may play a positive role in the treatment of hyperuricemia.

Our study still has some limitations. Firstly, the duration was relatively short, the long-term beneficial effects of HRW warrant further investigation. Secondly, the daily dietary and physical activity of the participants were not monitored. Thirdly, more biomarkers such as oxidative stress and inflammatory indicators were not addressed in the study. Moreover, due to the limited number of female participants in our study, we were unable to examine the potential impact of HRW on gender-based differences.

## 5 Conclusion

In conclusion, consumption of HRW at a dose of 330 mL each time and three times daily (totally 4.61 mg H_2_) for 8 weeks significantly reducing the blood uric acid level in patients with hyperuricemia. It is indicated that a long-term consumption of HRW might exert a beneficial effect in the management of uric acid level.

## Ethics statement

The trial was approved by the Ethical Committee of the Second Affiliated Hospital of Shandong First Medical University (No.2022-122) and was registered at the Chinese Clinical Trial Registry (chictr.org.cn) with the registration number ChiCTR2200066369 (registered on: December 2, 2022).

## Data availability statement

The data that support the findings of this study are available from the corresponding author upon reasonable request.

## CRediT authorship contribution statement

**Fenglin Wu:** Writing – original draft, Investigation, Formal analysis, Data curation. **Jun Ma:** Writing – original draft, Investigation, Data curation. **Junli Xue:** Writing – original draft, Investigation. **Xue Jiang:** Writing – original draft, Investigation. **Jinyu Liu:** Writing – original draft, Investigation. **Jiashuo Zhang:** Writing – original draft, Investigation. **Yazhuo Xue:** Writing – review & editing, Supervision, Investigation, Conceptualization. **Boyan Liu:** Writing – review & editing, Writing – original draft, Supervision, Investigation, Funding acquisition, Formal analysis, Conceptualization. **Shucun Qin:** Writing – review & editing, Investigation, Funding acquisition, Conceptualization.

## Declaration of competing interest

None.
