# Supplementary material for: Effects of hydrogen-rich water on blood uric acid in patients with hyperuricemia: A randomized placebo-controlled trial
Source: Heliyon. 2024 Aug 15;10(16):e36401. doi: 10.1016/j.heliyon.2024.e36401 (PMC11385766; doi:10.1016/j.heliyon.2024.e36401)
Supplement: Multimedia component 1 [file mmc1.docx]

**Table S1.** The blood uric acid levels and the changes in blood uric acid compared to the baseline

|  | **Placebo group**  **(*n* = 32)** | **Change in uric acid from baseline** | **Low-HRW group**  **(*n* = 35)** | **Change in uric acid from baseline** | **High-HRW group**  **(*n* = 33)** | **Change in uric acid from baseline** |
| --- | --- | --- | --- | --- | --- | --- |
| Baseline | 486.4 ± 71.1 |  | 484.8 ± 64.6 |  | 488.2 ± 54.1 |  |
| 4 weeks | 511.9 ± 91.1 | 25.3 (-66.0 to 185) | 485.7 ± 82.4 | 0.69 (-19.1 to 20.4) | 481.6 ± 81.1 | -6.74 (-35.0 to 21.5) |
| 8 weeks | 487.2 ± 84.5 | 0.60 (-23.8 to 24.8) | 466.0 ± 97.4 | -19.0 (-44.0 to 6.10) | 446.8 ± 57.1* | -41.6 (-71.0 to -12.3) |

Data are mean ± SD. * *P* < 0.05 compared to the baseline.
